# Including unpublished surveys in reviews on Chagas disease in Mexico

**DOI:** 10.1186/s40985-020-00140-7

**Published:** 2020-11-11

**Authors:** Pierre Buekens, Jorge López-Cárdenas, Eric Dumonteil, Nicolas Padilla-Raygoza

**Affiliations:** 1 School of Public Health and Tropical Medicine, Tulane University, 1440 Canal St., Suite 2001, New Orleans, LA 70112 USA; 2 Public Health State Laboratory of the State of Guanajuato, Leon, Guanajuato, Mexico; 3 School of Medicine, University of Celaya, Celaya, Guanajuato, Mexico

**Keywords:** Chagas disease, Guanajuato, Mexico, *Trypanosoma cruzi*

## Abstract

A consequence of the late awareness of Chagas disease in North America is that many early studies were never published in peer-reviewed journals and are not easily accessible for inclusion in systematic reviews. We reviewed data from the state of Guanajuato, Mexico, as an illustration. Three population-based surveys have been performed between 1991 and 2002 and were never fully published. Systematic reviews should recognize this publication bias.

## Background

Chagas disease or American trypanosomiasis is a major cause of cardiac disease in the Americas [1]. Most of the efforts to control Chagas disease have been focused on South and Central America, while the awareness of Chagas disease is more recent in North America, including Mexico [2]. A recent systematic review of Mexican population-based data from 2006 to 2017 estimated the *Trypanosoma cruzi* national seroprevalence at 3.4% [1]. Historically, Mexico has been divided into “endemic” and “non-endemic” areas for Chagas disease [3]. There is now a growing consensus that Chagas disease is a national problem in Mexico, regardless of the federal entity.

A consequence of the late awareness of Chagas disease in North America is that many early studies were never published in peer-reviewed journals and are not easily accessible for inclusion in systematic reviews. We will review data from the state of Guanajuato as an illustration. The state of Guanajuato is north of Mexico City and is one of the states sending more immigrants to the USA [4]. Detailed entomological studies have shown that the vector is present all over the state [5, 6]. Three population-based surveys have been performed between 1991 and 2002 and were never fully published. A serological survey performed by Juárez Leyva in 1991 in San José de la Presa, Purísima del Rincón, Guanajuato, found a *T*. *cruzi* seroprevalence of 6.1% (*n* = 228) [5, 7]. Another serological survey performed in Guanajuato in 1999–2000 in 60 communities found a seroprevalence of 2.6% (*n* = 1730) [5]. A serological survey performed in Ciudad Manuel Doblado, Guanajuato, in 2002, found 2.0% seroprevalence among 200 children and adolescents < 18 years old [7]. Studies performed more recently were published and showed seroprevalence of 0.8% in Celaya, Guanajuato, in 2006–2007 and of 3.8% in León, Guanajuato, in 2014–2015 [8, 9]. The reported number of cases of Chagas disease from Guanajuato increased over time, suggesting a growing awareness of the disease [10].

## Conclusion

Many studies on Chagas disease in Mexico were most likely not published in peer-reviewed journals. Systematic reviews should recognize this publication bias and search for unpublished data as much as possible.

## Data Availability

Not applicable.
